# Uses of equipoise in discussions of the ethics of randomized controlled trials of COVID-19 therapies

**DOI:** 10.1186/s12910-021-00712-5

**Published:** 2021-10-21

**Authors:** Hayden P. Nix, Charles Weijer

**Affiliations:** 1 Schulich School of Medicine and Dentistry, Western University, 1151 Richmond St, London, ON N6A 5C1 Canada; 2 Department of Medicine, Epidemiology and Biostatistics, and Philosophy, Western University, London, ON Canada

**Keywords:** Research ethics, COVID-19, Equipoise, Randomised controlled trial

## Abstract

**Background:**

Early in the COVID-19 pandemic, the urgent need to discover effective therapies for COVID-19 prompted questions about the ethical problem of randomization along with its widely accepted solution: equipoise. In this scoping review, uses of equipoise in discussions of randomized controlled trials (RCT) of COVID-19 therapies are evaluated to answer three questions. First, how has equipoise been applied to COVID-19 research? Second, has equipoise been employed accurately? And third, do concerns about equipoise pose a barrier to the ethical conduct of COVID-19 RCTs?

**Methods:**

Google Scholar and Pubmed were searched for articles containing substantial discussion about equipoise and COVID-19 RCTs. 347 article titles were screened, 91 full text articles were assessed, and 48 articles were included. Uses of equipoise were analyzed and abstracted into seven categories.

**Results and discussion:**

Approximately two-thirds of articles (33/48 articles) used equipoise in a way that is consistent with the concept. They invoked equipoise to support (1) RCTs of specific therapies, (2) RCTs in general, and (3) the early termination of RCTs after achieving the primary outcome. Approximately one-third of articles (15/48 articles) used equipoise in a manner that is inconsistent with the concept. These articles argued that physician preference, widespread use of unproven therapies, patient preference, or expectation of therapeutic benefit may undermine equipoise and render RCTs unethical. In each case, the purported ethical problem can be resolved by correcting the use of equipoise.

**Conclusions:**

Our findings highlight the continued relevance of equipoise as it supports the conduct of well-conceived RCTs and provides moral guidance to physicians and researchers as they search for effective therapies for COVID-19.

**Supplementary Information:**

The online version contains supplementary material available at 10.1186/s12910-021-00712-5.

## Background

The devastating impact of COVID-19 has been felt around the world. Early in the pandemic, as cases and deaths climbed, there was an urgent need to discover effective therapies. Randomized controlled trials (RCT) offer a rigorous method to evaluate potential therapies, but the urgency of the pandemic prompted questions about how such trials could be conducted ethically. Historically, RCTs have raised the ethical problem of randomization: how can a physician uphold her duty of care while allocating treatments at random [1]? The COVID-19 pandemic brought this question to the fore once again.

Equipoise is widely regarded as a compelling solution to the ethical problem of randomization [1]. Benjamin Freedman developed the concept of equipoise in 1987 [2]. According to Freedman, equipoise is a state of honest, professional disagreement in the community of expert practitioners as to the preferred treatment for a condition [2]. Central to equipoise is the idea that competent care is defined by the expert community, and not the opinion of an individual practitioner. Equipoise holds that an RCT may be initiated ethically if there is a lack of evidence or conflicting evidence regarding the treatment(s) or intervention(s) in question. As a result, equipoise supports the evaluation of routinely used treatment when evidence of its efficacy is lacking. During the course of an RCT, equipoise is disrupted if the evidence in favour of one treatment becomes so strong that “no open-minded clinician informed of the results” would favour the inferior treatment arm [2]. If this occurs, the RCT ought to be terminated.

Since its inception, equipoise has remained a key concept in research ethics and its scope of application has expanded. Equipoise now encompasses innovative RCT designs, such as cluster randomised trials and adaptive platform trials [3]. Further, it encompasses trials of interventions that are outside of the doctor-physician relationship. For example, MacKay argues that, in trials of public policy interventions, the community of public policy experts must be in a state of honest, professional disagreement about the merits of the trial interventions [4]. With these expansions in scope, the core of the concept of equipoise remains intact. Early in the pandemic, the urgent need for COVID-19 therapies posed a new challenge for equipoise.

In this article, we explore the uses of the concept of equipoise in ethical discussions of RCTs of COVID-19 therapies. We ask three questions. First, how has equipoise been applied to COVID-19 research? Second, has equipoise been employed accurately? And third, do concerns about equipoise pose a barrier to the ethical conduct of RCTs?

## Methods

The search terms “equipoise” and “COVID-19,” “SARS-CoV-2,” or “coronavirus,” were input into Google Scholar and Pubmed to identify articles containing a substantial discussion about the application of equipoise to COVID-19 therapeutic trials. These databases were chosen to include both peer-reviewed and grey literature. The search was limited to January 2020–June 2020 to focus on the period in the pandemic in which there were no evidence-based treatments for COVID-19. As such, all articles were written prior to the publication of the RCTs that established remdesivir and dexamethasone as effective treatments for COVID-19.

Article titles were screened to identify articles about COVID-19 research. Next, full texts were searched for the term equipoise. Articles were included if they contained a substantial discussion about the application of equipoise to COVID-19 therapeutic trials. Articles were excluded if equipoise was (1) used ambiguously; (2) applied to research for diseases other than COVID-19; (3) used with an alternative denotation (e.g., the term “physiological equipoise” refers to a state of homeostasis in the body); (4) applied to clinical care for diseases other than COVID-19; (5) solely in the reference list; or (6) applied to pre-clinical research. Included full text articles were searched for the term equipoise, the surrounding text was reviewed, and the authors’ use of the concept of equipoise was categorized. Ambiguous quotes were discussed, and consensus categorizations were reached in all cases.

## Results

**Fig. 1:**
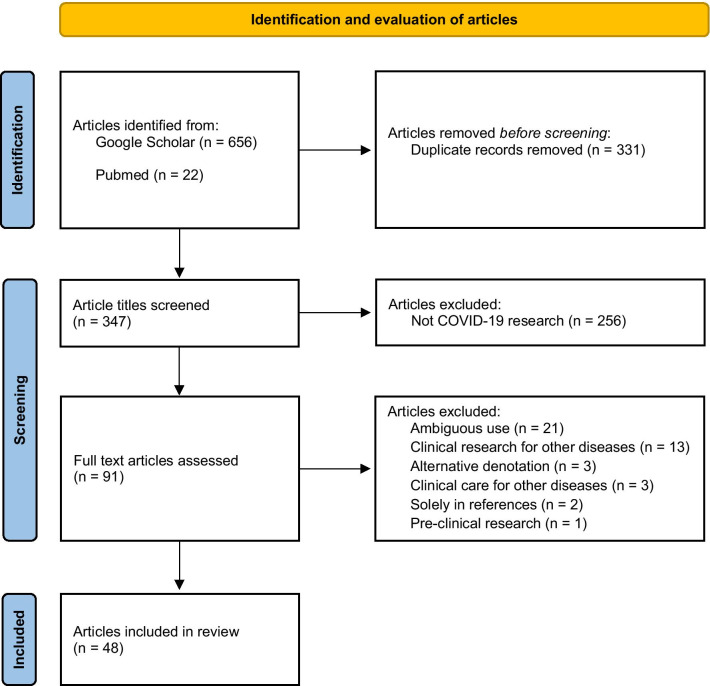
Study flow diagram

The search yielded 678 records. Duplicate records were excluded, yielding a total of 347 articles. Title screening excluded 256 articles that were not about COVID-19 research. Ninety-one full text articles were assessed. Of the excluded articles, 21 used equipoise ambiguously; 13 applied it research for diseases other than COVID-19; 3 used the term equipoise with an alternative denotation; 2 applied it to clinical care for diseases other than COVID-19; 2 used it only in the reference list; and 1 applied it to pre-clinical research. Forty-seven articles were included and analyzed (Fig. 1). One article used equipoise twice and was therefore counted and categorized twice.

Table 1 summarizes the uses of equipoise in discussions of the ethics of RCTs of COVID-19 therapy (for Table 1 references, see Additional file 1). Categories are listed from most to least frequent.

**Table 1:** Uses of equipoise in articles discussing the ethics of randomized controlled trials of COVID-19 therapies

Category of use		Examples	Articles in which theme is found (See: Additional file 1)
1	Equipoise supports the conduct of RCTs for a specific treatment	“Given that ruxolitinib clearly reduces antiviral immunity, the rationale to test its use in treating patients with severe or very severe COVID-19 illness merits at least equipoise or serious reconsideration” [5] “We believe that clinical equipoise regarding risk/benefit to the individual patient justifies well the conduct of this [azithromycin] RCT” [15]	Alexander et al Betts and Young Christensen et al Duska et al Dzik El Rhazi and Adarmouch Hall et al Ingraham et al Kazi et al Keshtkar-Jahroni and Bavari Maraj et al McNicholas et al Mehta et al Prasad et al Ramacciotti et al Sarzani et al Schilling et al Singh et al Spivak and Hess
2	Equipoise supports the conduct of therapeutic RCTs for COVID-19 in general	“As clinicians caring for patients dying from COVID-19, we too yearn for a novel therapy for this novel disease. We also recognise and appreciate the scientific value of expert observations. Indeed, they are crucial to identify aspects of management where there truly is equipoise and thus indication for rigorous study” [6] “We must reason critically and reflect on the biases that may influence our thinking processes, critically appraise evidence in deciding how to treat patients, and use anecdotal observations only to generate hypotheses for trials that can be conducted with clinical equipoise” [16]	Aronson et al Chen and Enache Eyal and Lipsitch Galloway et al Haushofer and Metcalf Kalil London and Kimmelman Monrad Moores et al Pulley et al Rose et al Yusuf and Maiwald Zagury-Orly and Schwartzstein
3	Equipoise is disrupted by physician preference, threatening researchers’ ability to conduct RCTs	“Should care providers' equipoise falter before the end of the study, they may be strongly tempted to ignore subsequent treatment assignments” [7] “But many clinicians are not able to maintain such equipoise in the face of catastrophe. Therefore, I propose an approach to consideration of bedside implementation of unproven therapies for life-threatening COVID-19 for comment and criticism” [17]	Alderighi and Rasoini Angus Corral-Gudino et al Grobler et al Magaret et al Raschke
4	Equipoise is disrupted by the widespread use of treatments for COVID-19, threatening researchers’ ability to conduct RCTs	“This data-free approach will ultimately harm more patients than it helps, as one-off administration of medications ruins clinical equipoise about their use” [18] “Another serious problem with routine use of unproven agents for SARS-CoV-2 is that clinical equipoise is lost and an experimental agent becomes de facto standard of care, potentially seriously compromising the ability to do placebo-controlled trials” [8]	Carley et al Ramnath, Zar et al Ramnath, McSharry et al Singer Waterer et al
5	Equipoise is disrupted by patient preference, threatening researchers’ ability to conduct RCTs	“If recruitment is difficult because of placebo-arm aversion, this should be a signal as to the study’s lack of equipoise” [19]	Alderighi and Rasoini Keane Veatch
6	Equipoise is disrupted if there is too great an expectation of benefit prior to trial onset, threatening researchers’ ability to conduct RCTs	“Therefore, the drugs or interventions that are planned for RCT may already be expected to work, although they have no concrete evidence of efficacy yet. In this regard, RCTs that start with such a premise can hardly be seen as truly adhering to the ‘principle of clinical equipoise’” [10]	Lee et al
7	Equipoise is disrupted if the primary endpoint in an RCT reaches statistical significance in an interim analysis	“The NASEM committee supported RCTs at the outset of the Ebola outbreak because it was unknown whether any agents would be safe and effective; true equipoise existed between the experimental treatment and placebo. Thus, the use of placebo in the ACCT-1 trial was warranted based on established scientific and ethical grounds. However, at the point when NIAID stopped the ACCT-1 trial, it would be difficult to say that there was no effective agent in order to justify the continued use of placebo in ACCT-1 or in the adaptive clinical trials designs that will follow ACCT-1” [11]	Mozersky et al

In the first category, equipoise is invoked to justify the conduct of an RCT of a particular COVID-19 therapy or class of therapy (19 articles). Articles assessed the evidence for novel therapies, such as mechanism of action, animal studies, and human studies, to evaluate whether it supports the conduct of an RCT. For instance, Betts and colleagues reviewed evidence supporting ruxolitinib, an interleukin-6 blocker, concluding that, “the rationale to test its use in treating patients with severe or very severe COVID-19 illness merits at least equipoise or serious reconsideration,” and that “disciplined clinical research” is justified [5].

In the second category, equipoise is invoked to justify the conduct of RCTs in general (13 articles), citing the lack of evidence-based treatment for COVID-19. For example: “We…recognise and appreciate the scientific value of expert observations. Indeed, they are crucial to identify aspects of management where there truly is equipoise and thus indication for rigorous study” [6].

In the third category, authors assert that physician preference may disrupt equipoise, and thereby threaten researchers’ ability to conduct RCTs ethically (6 articles). For example, Magaret and colleagues write, “Should care providers' equipoise falter before the end of the study, they may be strongly tempted to ignore subsequent treatment assignments” [7].

In the fourth category, authors argue that the widespread use of therapies for COVID-19 may disrupt equipoise, and thereby threaten researchers’ ability to conduct RCTs ethically (5 articles). For instance, Waterer and colleagues argue that a “serious problem with routine use of unproven agents for SARS-CoV-2 is that clinical equipoise is lost and an experimental agent becomes de facto standard of care” [8].

In the fifth category, authors argue that patient preference may disrupt equipoise, and thereby threaten researchers’ ability to conduct RCTs ethically (3 articles). For instance, Veatch says, “a patient may have a preference for one arm while researchers are legitimately and honestly indifferent” and this may disrupt equipoise [9].

In the sixth category, authors argue that an expectation of benefit may disrupt equipoise, and thereby threaten researchers’ ability conduct of RCTs ethically (1 article). The idea is that if there is an expectation that a novel therapy for COVID-19 will benefit patients, equipoise may be disrupted. Lee and colleagues argue that “drugs or interventions that are planned for RCT may already be expected to work, although they have no concrete evidence of efficacy yet. In this regard, RCTs that start with such a premise can hardly be seen as truly adhering to the ‘principle of clinical equipoise’” [10].

In the seventh and final category, authors invoke equipoise to justify stopping an RCT when a statistically significant difference in the primary outcome measure occurs in an interim analysis (1 article). Stating that a placebo control was justified by equipoise at the beginning the trial, Mozersky and colleagues go on to claim that “at the point when NIAID stopped the ACCT-1 trial, it would be difficult to say that there was no effective agent in order to justify the continued use of placebo in ACCT-1” [11].

## Discussion

Reassuringly, over two-thirds of articles (33/48 articles) invoke equipoise in ways that are consistent with the concept. This includes the first, second, and seventh categories. In each case, authors correctly use equipoise to support the ethical conduct of RCTs.

The first and second categories use equipoise to argue in support of conducting RCTs to evaluate unproven COVID-19 therapies, both specifically and in general. Articles correctly indicate that equipoise hinges on evidence and supports the initiation of an RCT to evaluate a novel therapy when there exists a plausible rationale and definitive evidence of efficacy is lacking.

In the seventh category, equipoise is used to justify the early termination of the placebo arm in ACCT-1, an adaptive RCT that evaluated *inter alia* the efficacy of remdesivir for COVID-19 [11]. They argue that the achievement of a statistically significant difference in the primary outcome of the trial (time to recovery) was sufficient evidence to disrupt equipoise. Recall, accumulating evidence is sufficient to disrupt equipoise when “no open-minded clinician informed of the results” would favour the inferior treatment arm [2]. Mozersky and colleagues’ appeal to equipoise is in accordance with this guidance and is therefore sound.

Approximately one-third of articles (15/48 articles) use equipoise in ways that are inconsistent with the concept. In the third, fourth, fifth, and sixth categories, authors claim that equipoise may be disrupted by physician preference, widespread use, patient preference, and expectation of benefit, respectively, and that the risk of disrupting equipoise poses a barrier to the ethical conduct of RCTs for COVID-19 therapies. If correct, these concerns seem to threaten the ethical permissibility of this important research. This, in turn, could slow its progress and cause moral distress among physicians and researchers. But can these factors disrupt equipoise?

Can physician preference disrupt equipoise? It cannot, because equipoise refers to uncertainty in the community of expert practitioners and is not disrupted when an individual practitioner has a treatment preference. A physician may be of the opinion that a novel COVID-19 therapy works, but professionalism demands that she recognize when evidence has yet to be gathered to establish the therapy’s efficacy. Freedman suggests that when this occurs the physician ought to disclose her treatment preference during the consent process, along with emphasis “that this preference is not shared by others” [2].

Can widespread use of a therapy disrupt equipoise? It cannot, because equipoise depends on the evidence of efficacy and is not undermined by widespread use of an intervention when rigorous evidence of efficacy is lacking. Widespread use may lead to shortages of the study drug, physician reluctance to enroll sick patients, and patient refusal of consent. These factors may practically impede researchers’ ability to conduct an RCT. However, none of this speaks to equipoise or the ethics of the RCT. An instructive example is Moseley and colleagues’ placebo controlled RCT of arthroscopic lavage of the knee [12]. Hey and colleague argue that despite decades-long use of arthroscopic lavage, equipoise supports the conduct of a placebo-controlled trial “when the effectiveness of the standard of care has been called into question…[by] doubts about the supporting body of existing evidence” [13].

Can patient preference disrupt equipoise? It cannot, because equipoise is distinct from the ethics of consent [2]. The problem of randomization asks how the physician’s duty of care to the patient can be consistent with allocating treatment to the patient at random. Equipoise solves this problem by pointing out that randomization aligns with the duty of care when the community of practitioners is uncertain as to the preferred treatment. If there is equipoise, it matters not from an ethical standpoint if a patient has a treatment preference. The patient has the freedom to accept or decline enrollment, but neither decision throws equipoise or the ethics of the trial into question.

Can an expectation of benefit disrupt equipoise? In exceptional cases, it can. While the RCT is a rigorous method, it is not always required. In rare instances, evidence from an uncontrolled trial may be sufficient if the treatment effect is large and patient outcome without treatment is predictable. For example, Pasteur’s rabies vaccine allowed most patients to survive rabies, an otherwise nearly uniformly fatal infection [14]. RCTs are generally required because most medical interventions have small or medium effects and patients may improve without treatment. Thus, while preliminary indications of efficacy in uncontrolled trials of therapies for COVID-19 may justify evaluating the intervention in an RCT, such evidence generally does not undermine equipoise.

In the third, fourth, fifth, and sixth categories of use, an incorrect understanding of equipoise led authors to conclude that equipoise poses a barrier to the conduct of well-designed RCTs of COVID-19 therapies. These misconceptions are problematic because they could unnecessarily slow the progress of this important research and cause moral distress among physicians and researchers conducting these RCTs.

Correcting these misconceptions reveals that equipoise supports the conduct of well-conceived RCTs of COVID-19 therapies. Equipoise refers to uncertainty in the community of practitioners, not individual physicians; equipoise depends on the evidence for a treatment, not its prevalence of use; patient preference is a matter of consent, not equipoise; and, barring rare exceptions, expectations of benefit support equipoise rather than undermine it. Correctly applying equipoise promotes the conduct of well-conceived RCTs of COVID-19 therapies and may diminish moral distress among physicians and researchers involved in these trials.

This study has several limitations. First, using the search term “equipoise” excluded articles that discuss the concept of equipoise without explicitly using the term. Second, this methodology fails to provide insight into how research ethics committees applied equipoise when reviewing RCTs of COVID-19 therapies. Future work is required to describe and assess the use of equipoise in research ethics committee review of RCTs of COVID-19 therapies.

## Conclusions

The urgency of pandemic understandably prompted concerns about the ethical conduct of COVID-19 RCTs. In this brief report, we asked: how has equipoise been applied to COVID-19 research? Has equipoise been employed accurately? And does equipoise support the conduct of COVID-19 RCTs?

Most articles applied equipoise to support the ethical initiation and termination of COVID-19 RCTs; this use was consistent with the concept. A minority of articles used equipoise to raise concerns about the ethical conduct of COVID-19 RCTs. These uses were inconsistent with equipoise; in each case, correcting the erroneous equipoise claim removed the alleged barrier. When employed correctly, equipoise supports the conduct of well-conceived RCTs, providing moral guidance to physicians and trialists as they search for effective therapies for COVID-19.

## Supplementary Information

**Additional file 1.** References for abstracted articles.

## Data Availability

All data generated or analysed during this study are included in this published article [and its supplementary information files].

## Abbreviation

RCT: Randomized controlled trial
