# Supplementary material for: Uses of equipoise in discussions of the ethics of randomized controlled trials of COVID-19 therapies
Source: BMC Med Ethics. 2021 Oct 21;22:143. doi: 10.1186/s12910-021-00712-5 (PMC8528943; doi:10.1186/s12910-021-00712-5)
Supplement: Supplementary file 1 — Additional file 1. References for abstracted articles. [file 12910_2021_712_MOESM1_ESM.doc]

**ADDITIONAL FILE 1**

**Table 1 References**

| Category of use | | References |
| --- | --- | --- |
| 1 | Equipoise supports the conduct of RCTs for a specific treatment | Alexander P, Debono V, Mammen M, et al. COVID-19 coronavirus research has overall low methodological quality thus far: case in point for chloroquine/hydroxychloroquine. J Clin Epidemiol 2020;123:120–6. doi:10.1016/j.jclinepi.2020.04.016  Betts BC, Young JW. Less Can Be More When Targeting Interleukin-6-Mediated Cytokine Release Syndrome in Coronavirus Disease 2019. Crit Care Explor 2020;2:e0138. doi:10.1097/cce.0000000000000138  Christensen HR, Christensen R. Effectiveness of Interleukin-6 Receptor Inhibitors in the Management of Patients with Severe SARS-CoV-2 Pneumonia: An Open-Label, Multicenter Sequential and Cluster Randomized Trial Background. 2020. www.sst.dk/corona (accessed 20 Jul 2020)  Duška F, Waldauf P, Halačová M, et al. Azithromycin added to hydroxychloroquine for patients admitted to intensive care due to coronavirus disease 2019 (COVID-19) - protocol of randomised controlled trial AZIQUINE-ICU. Trials 2020;21:631. doi:10.1186/s13063-020-04566-x  Dzik S. COVID-19 convalescent plasma: now is the time for better science. Transfus Med Rev Published Online First: 2020. doi:10.1016/j.tmrv.2020.04.002  El Rhazi K, Adarmouch L. Ethical issues related to the hydroxychloroquine treatment prescription for Covid-19. Ethics, Med Public Heal 2020;14:100547. doi:10.1016/j.jemep.2020.100547  Hall MW, Joshi Phd I, Leal Dpm L, et al. Immune modulation in COVID-19: Strategic considerations for personalized therapeutic intervention. Clin Infect Dis 2020;ciaa904. doi:10.1093/cid/ciaa904/5865455  Ingraham N, Barakat A, Reilkoff R, et al. Understanding the renin-angiotensin-aldosterone-SARS-CoV-axis: a comprehensive review. Eur Respir J Published Online First: 2020. doi:10.1183/13993003.00912-2020  Kazi S, Malinowski AK, Othman M. The delights and perils of publishing, knowledge-sharing and critique during a pandemic: Observations from COVID-19 coagulopathies. Thromb Res 2020;192:37–9. doi:10.1016/j.thromres.2020.05.023  Keshtkar-Jahromi M, Bavari S. A call for randomized controlled trials to test the efficacy of chloroquine and hydroxychloroquine as therapeutics against novel coronavirus disease (COVID-19). Am J Trop Med Hyg 2020;102:932–3. doi:10.4269/ajtmh.20-0230  Maraj I, Hummel JP, Taoutel R, et al. Incidence and determinants of QT interval prolongation in COVID-19 patients treated with hydroxychloroquine and azithromycin. J Cardiovasc Electrophysiol 2020;31:1904–7. doi:10.1111/jce.14594  McNicholas B, Cosgrave D, Giacomini C, et al. Prone positioning in COVID-19 acute respiratory failure: just do it? Br J Anaesth 2020;in press. doi:10.1016/j.bja.2020.06.003  Mehta P, Porter JC, Manson JJ, et al. Therapeutic blockade of granulocyte macrophage colony-stimulating factor in COVID-19-associated hyperinflammation: challenges and opportunities. Lancet Respir Med 2020;8:822–30. doi:10.1016/S2213-2600(20)30267-8  Prasad A, Panhwar S, Hendel RC, et al. COVID-19 and the cardiovascular system: A review of current data, summary of best practices, outline of controversies, and illustrative case reports. Am Heart J 2020;226:174–87. doi:10.1016/j.ahj.2020.06.009  Ramacciotti E, Macedo AS, Biagioni RB, et al. Evidence-Based Practical Guidance for the Antithrombotic Management in Patients With Coronavirus Disease (COVID-19) in 2020. Clin Appl Thromb 2020;26:1–8. doi:10.1177/1076029620936350  Sarzani R, Giulietti F, Di Pentima C, et al. Disequilibrium between the classic renin-angiotensin system and its opposing arm in SARS-CoV-2-related lung injury. Am Jounral Physiol Lung Cell Mol Physiol 2020;319:L325–36. doi:10.1152/ajplung.00189.2020  Schilling W, Taylor W, Ashley E. Chloroquine/ hydroxychloroquine prevention of coronavirus disease (COVID-19) in the healthcare setting; a randomised, placebo-controlled prophylaxis study (COPCOV). ClinicalTrials.gov. 2020. https://www.tropmedres.ac/files/moru-bangkok-files/copcov-protocol-version-4-0-dated-09-apr-20.pdf  Singh A, Singh A, Singh R, et al. Remdesivir in COVID-19: A critical review of pharmacology, pre-clinical and clinical studies. Diabetes Metab Syndr Clin Res Rev 2020;14:641–8. doi:10.1016/j.dsx.2020.05.018  Spivak A, Hess R. CLINICAL STUDY PROTOCOL: Hydroxychloroquine for Outpatients with Confirmed COVID-19 (HCQ Trial). 2020. https://icts.uci.edu/funding/funding material /COVID.pdf |
| 2 | Equipoise supports the conduct of therapeutic RCTs for COVID-19 | Aronson J, DeVito N, Ferner R, et al. The ethics of COVID-19 treatment studies: too many are open, too few are double-masked. Cent. Evidence-Based Med. 2020.https://www.cebm.net/covid-19/the-ethics-of-covid-19-treatment-studies-too-many-are-open-too-few-are-double-masked/ (accessed 20 Jul 2020).  Chen Y, Enache B. Every COVID-19 patient should be able to join a randomised clinical trial. LSE COVID-19 Blog. 2020.https://blogs.lse.ac.uk/covid19/2020/06/02/every-covid-19-patient-should-be-able-to-join-a-randomised-clinical-trial/ (accessed 20 Jul 2020).  Eyal N, Lipsitch M. Opinion: It’s ethical to test promising coronavirus vaccines against less-promising ones. Proc Natl Acad Sci U S A 2020;117:18898–901. doi:10.1073/pnas.2014154117  Galloway J, Norton S, Barker R, et al. A clinical risk score to identify patients with COVID-19 at high risk of critical care admission or death: An observational cohort study. J Infect 2020;81:282–8. doi:10.1016/j.jinf.2020.05.064  Haushofer J, Metcalf C. Which interventions work best in a pandemic? Science (80- ) 2020;368:1063–5. doi:10.1126/science.abb6144  Kalil A. Treating COVID-19—off-label drug use, compassionate use, and randomized clinical trials during pandemics. J Am Med Assoc 2020;323:1897–8. doi:10.1001/jama.2020.4742  London AJ, Kimmelman J. Against pandemic exceptionalism. Science (80- ) 2020;386:476–7. doi:10.1126/science.abc1731  Monrad JT. Ethical considerations for epidemic vaccine trials. J Med Ethics 2020;46:465–9. doi:10.1136/medethics-2020-106235  Moores L, Tritschler T, Brosnahan S, et al. Prevention, diagnosis and treatment of venous thromboembolism in patients with COVID-19: CHEST Guideline and Expert Panel Report. Chest 2020;158:1143–63. doi:10.1016/j.chest.2020.05.559  Pulley JM, Jerome RN, Rice TW, et al. Equipoise and research in the current COVID-19 pandemic. J Clin Transl Sci 2020;20:1–2. doi:10.1017/cts.2020.48  Rose MR, Hiltz KA, Stephens RS, et al. Novel viruses, old data, and basic principles: how to save lives and avoid harm amid the unknown. Lancet Respir Med 2020;8:661–3. doi:10.1016/S2213-2600(20)30236-8  Yusuf E, Maiwald M. COVID-19, equipoise and observational studies: a reminder of forgotten issues. Infection Published Online First: 2020. doi:10.1007/s15010-020-01466-9  Zagury-Orly I, Schwartzstein R. Covid-19—a reminder to reason. N Engl J Med 2020;383:e12. doi:10.1056/NEJMp2009405 |
| 3 | Equipoise is disrupted by physician preference, threatening researchers’ ability to conduct RCTs. | Alderighi C, Rasoini R. The naked king in the pandemic: About the production and communication of scientific knowledge at the time of SARS-CoV-2. Recenti Prog Med Published Online First: 2020. doi:10.1701/3407.33920  Angus D. Optimizing the trade-off between learning and doing in a pandemic. J Am Med Assoc 2020;323:1895–6. doi:10.1001/jama.2020.4984  Corral-Gudino L, Bahamonde A, Arnaiz-Revillas F, et al. GLUCOCOVID: A controlled trial of methylprednisolone in adults hospitalized with COVID-19 pneumonia. medRxiv Prepr Published Online First: 2020. doi:10.1101/2020.06.17.20133579  Grobler C. COVID-19: Mental health and clinical equipoise in the face of moral injury. South African J Bioeth Law 2020;13:21–2. doi:10.7196/SAJBL.2020.v13i1.724  Magaret AS, Jacob ST, Halloran ME, et al. Multigroup, Adaptively Randomized Trials Are Advantageous for Comparing Coronavirus Disease 2019 (COVID-19) Interventions. Ann Intern Med Published Online First: 2020. doi:10.7326/M20-2933  Raschke R. Choosing among unproven therapies for the treatment of life-threatening covid-19 infection: a clinician’s opinion from the beside. Southwest J Pulm Crit Care 2020;20:131–4. doi:10.13175/swjpcc026-20 |
| 4 | Equipoise is disrupted by the widespread use of treatments for COVID-19, threatening researchers’ ability to conduct RCTs | Carley S, Horner D, Body R, et al. Evidence-based medicine and COVID-19: What to believe and when to change. Emerg Med J 2020;0:1–4. doi:10.1136/emermed-2020-210098  Ramnath VR, Zar HJ, Malhotra A. Addressing the “What do we have to lose? Just give the drug” rationale: making the case for clinical trials and against off-label use in COVID-19. J Thorac Dis 2020;12:3031–4. doi:10.21037/jtd-20-2011  Ramnath VR, McSharry DG, Malhotra A. Do No Harm: Reaffirming the Value of Evidence and Equipoise While Minimizing Cognitive Bias in the Coronavirus Disease 2019 Era. Chest 2020;158:873–6. doi:10.1016/j.chest.2020.05.548  Singer D. Health policy and technology challenges in responding to the COVID-19 pandemic. Heal Policy Technol 2020;9:123–5. doi:10.1016/j.hlpt.2020.04.011  Waterer GW, Rello J, Wunderink RG. COVID-19: First do no harm. Am J Respir Crit Care Med 2020;201:1324–5. doi:10.1164/rccm.202004-1153ED |
| 5 | Equipoise is disrupted by patient preference, threatening our ability to conduct RCTs | Alderighi C, Rasoini R. The naked king in the pandemic: About the production and communication of scientific knowledge at the time of SARS-CoV-2. Recenti Prog Med Published Online First: 2020. doi:10.1701/3407.33920  Keane M. Covid-19: Time to Rethink the Randomized Controlled Trial and Consider More Efficient and Ethical Ways of Gaining Clinical Knowledge. SSRN Electron J Published Online First: 2020. doi:10.2139/ssrn.3619876  Veatch RM. Clinical Trials vs. Right to Try: Ethical Use of Chloroquine for Covid-19. Hast. Cent. 2020.thehastingscenter.org/clinical-trials-vs-right-to-try-ethical-use-of-chloroquine-for-covid-19 (accessed 20 Jul 2020). |
| 6 | Equipoise is disrupted if there is too great an expectation of benefit prior to trial onset, threatening researchers’ ability to conduct RCTs | Lee J, Shin HW, Lee JY, et al. A Comprehensive Analysis of Clinical Trials in the COVID-19 Pandemic Era. Med 2020;56. doi:10.3390/medicina56060315 |
| 7 | Equipoise is disrupted if the primary endpoint in an RCT reaches statistical significance in an interim analysis. | Mozersky J, Mann DL, Dubois JM. Journal Pre-proof The National Institute of Allergy and Infectious Diseases Decision to Stop the Adaptive COVID-19 Treatment Trial (ACCT-1): On Solid Ethical and Scientific Ground. JACC Basic to Transl Sci Published Online First: 2020. doi:10.1016/j.jacbts.2020.05.002 |
